# Epstein-Barr Virus BGLF4 Kinase Retards Cellular S-Phase Progression and Induces Chromosomal Abnormality

**DOI:** 10.1371/journal.pone.0039217

**Published:** 2012-06-29

**Authors:** Yu-Hsin Chang, Chung-Pei Lee, Mei-Tzu Su, Jiin-Tarng Wang, Jen-Yang Chen, Su-Fang Lin, Ching-Hwa Tsai, Min-Jei Hsieh, Kenzo Takada, Mei-Ru Chen

**Affiliations:** 1 Graduate Institute and Department of Microbiology, College of Medicine, National Taiwan University, Taipei, Taiwan; 2 General Education Center, National Taipei University of Nursing and Health Sciences, Taipei, Taiwan; 3 National Institute of Cancer Research, National Health Research Institutes, Zhunan, Taiwan; 4 Department of Tumor Virology, Institute for Genetic Medicine, Hokkaido University, Sapporo, Japan; University of Hong Kong, Hong Kong

## Abstract

Epstein-Barr virus (EBV) induces an uncoordinated S-phase-like cellular environment coupled with multiple prophase-like events in cells replicating the virus. The EBV encoded Ser/Thr kinase BGLF4 has been shown to induce premature chromosome condensation through activation of condensin and topoisomerase II and reorganization of the nuclear lamina to facilitate the nuclear egress of nucleocapsids in a pathway mimicking Cdk1. However, the observation that RB is hyperphosphorylated in the presence of BGLF4 raised the possibility that BGLF4 may have a Cdk2-like activity to promote S-phase progression. Here, we investigated the regulatory effects of BGLF4 on cell cycle progression and found that S-phase progression and DNA synthesis were interrupted by BGLF4 in mammalian cells. Expression of BGLF4 did not compensate Cdk1 defects for DNA replication in *S. cerevisiae*. Using time-lapse microscopy, we found the fate of individual HeLa cells was determined by the expression level of BGLF4. In addition to slight cell growth retardation, BGLF4 elicits abnormal chromosomal structure and micronucleus formation in 293 and NCP-TW01 cells. In Saos-2 cells, BGLF4 induced the hyperphosphorylation of co-transfected RB, while E2F1 was not released from RB-E2F1 complexes. The E2F1 regulated activities of the cyclin D1 and ZBRK1 promoters were suppressed by BGLF4 in a dose dependent manner. Detection with phosphoamino acid specific antibodies revealed that, in addition to Ser780, phosphorylation of the DNA damage-responsive Ser612 on RB was enhanced by BGLF4. Taken together, our study indicates that BGLF4 may directly or indirectly induce a DNA damage signal that eventually interferes with host DNA synthesis and delays S-phase progression.

## Introduction

Epstein-Barr virus (EBV) is a human herpesvirus which infects most of the human population worldwide. EBV is the causal agent of infectious mononucleosis and is highly associated with human malignant diseases, such as Burkitt’s lymphoma, Hodgkin’s disease, nasopharyngeal carcinoma (NPC), T cell lymphoma and gastric carcinoma [1]. EBV becomes latent in long-lived B cells after primary infection. Following various stimuli, the latent virus can be reactivated through the expression of two immediate early transactivators, Zta and Rta, which turn on a cascade of lytic gene expression and EBV replication [2].

EBV has evolved various strategies to optimize the host cell environment for viral replication and virion production and achieve a sophisticated balance between the use of host and viral machineries during lytic replication. The expression of S-phase cyclins, cyclin E and cyclin A, is up-regulated and leads to an increase in the associated S-phase Cdk activity and RB hyperphosphorylation in cells replicating EBV. Inhibition of S-phase Cdk by chemical Cdk inhibitors blocks the expression of immediate early and early genes and prevents lytic cycle progression, suggesting that S-phase Cdk activity is required for efficient initiation of lytic EBV replication [3]. However, it is worth noting that cellular DNA replication is blocked at the G1/S boundary upon EBV lytic cycle induction [4]. It has been speculated that the S-phase-like environment may provide materials for viral DNA replication and competition from host DNA replication is blocked by viral factors. In addition, a DNA damage response (DDR) is elicited during lytic EBV replication by activation of the ATM checkpoint signaling pathway, which has been proved to be required for the progression of virus replication [5]. On the other hand, repetitive reactivation of EBV was shown to promote genomic instability and tumor progression in NPC cells, suggesting viral factors may affect cell growth control or cell cycle progression [6].

EBV BGLF4 is a proline-dependent Ser/Thr protein kinase, which belongs to the family of conserved herpesvirus protein kinases (CHPKs). CHPKs are encoded by all herpesviruses and include UL13 of HSV-1, UL97 of HCMV, BGLF4 of EBV, U69 of human herpesvirus 6 (HHV6) and ORF36 of Kaposi sarcoma-associated herpesvirus (KSHV) [7], [8]. CHPKs are highly conserved with Cdks in the catalytic domain and were first shown to phosphorylate target sites of Cdk1 of cellular elongation factor 1delta (EF-1δ) [9]. As a result, there is the possibility that CHPKs may mimic Cdk1 activity in virus-infected cells at certain stages [7], [8]. A number of substrates of Cdk1 were later shown to be phosphorylated by BGLF4, including p27^KIP1^, MCM4, condensin, Topo II, lamin A/C. BGLF4 phosphorylates and induces the degradation of Cdk inhibitor p27^KIP^ upon lytic EBV replication, contributing to the establishment of an S-phase cellular environment, which is favorable for viral replication [10]. BGLF4 was shown to inactivate the MCM4-6-7 complex-associated DNA helicase through MCM4 phosphorylation and slow down the growth of HeLa cells, indicating that BGLF4 may have an inhibitory effect on host DNA replication [11]. Previously, we found that BGLF4 alone can induce cellular chromosome condensation through phosphorylation of condensin and activation of Topoisomerase II (Topo II) [12]. Moreover, BGLF4 participates in the regulation of efficient nuclear egress through phosphorylation of lamin A/C and disassembly of the nuclear lamina, mimicking Cdk1 activity at prophase [13]. However, BGLF4 actually has a broader substrate specificity than Cdk1. In an EBV-protein array phosphorylation assay, 10 of the 21 BGLF4 substrates were found to be substrates for Cdk1/cyclin B, while 10 of 12 Cdk1/cyclin B substrates were BGLF4 substrates [14]. Our previous study also pointed out that BGLF4 targets more residues on lamin A/C than does Cdk1 [13]. Conceivably, because BGLF4 activity is not inhibited by Cdk inhibitor, BGLF4 may be involved in more cellular regulation than Cdk1 [10], [13]. On the other hand, the Cdk-like function of CHPKs was examined recently by another group. It was found that beta- and gamma-, but not alpha-, CHPKs, share Cdk-like activity and phosphorylate RB at the Cdk4 and Cdk2 targeting residues, Ser780, 807 and Thr821, and promote bud growth in temperature-sensitive mutant yeasts in a compensation assay [15]. It was proposed by the authors that BGLF4 may also possesses Cdk-2 like activity to promote S-phase progression through RB phosphorylation [15].

In addition to Cdk-like activity, BGLF4 alone is capable of inducing H2AX phosphorylation in the absence of other viral proteins or agents inducing DNA damage [16]. More recently, BGLF4 was shown to phosphorylate and activate TIP60, a histone acetyltransferase regulating chromatin remodeling and responsive to DDR [17]. The BGLF4-mediated activation of TIP60 is required for autophosphorylation of ATM and expression of lytic genes, suggesting BGLF4 plays an essential role in the upstream process of DNA damage signaling, to facilitate viral replication.

Here we aimed to clarify the contradictory observations regarding BGLF4-induced phenomena and to determine the effects of BGLF4 on cell growth and cell cycle control in mammalian and yeast systems. The outcome of the BGLF4 kinase activity on cellular chromosome stability also was monitored. We show that BGLF4-elicited RB hyperphosphorylation does not lead to cell cycle progression or the activation of E2F1 responsive promoters. In addition to Ser780, BGLF4 induces the phosphorylation of RB at Ser612, which is an indication of DDR. In addition, BGLF4 elicits the formation of micronuclei in NPC-TW01 derived cell lines, suggesting that BGLF4 induces a DNA damage signal and causes the phosphorylation of RB at Ser612 that eventually interferes with host DNA synthesis and delays S-phase progression.

## Results

### BGLF4 Interferes with Cellular DNA Synthesis and S-phase Progression

In our previous studies, we found that BGLF4 induces prophase-like events, including unscheduled chromosome condensation and disassembly of nuclear lamina, independent of the cell cycle stage [12], [13]. In addition, BGLF4 was found to repress the activity of DNA helicase MCM4-6-7, implying that expression of BGLF4 does not favor cellular DNA synthesis [11]. In contrast, a recent study reported that EBV BGLF4 and its homologues in beta and gamma-herpesviruses elicit RB hyperphosphorylation and promote bud growth of a temperature-sensitive mutant yeast *cdc28-13*, suggesting BGLF4 has the potential to advance S-phase progression [15]. Here, we aimed to clarify the paradoxical effects of BGLF4 on cell growth and cell cycle progression.

**Figure 1:**
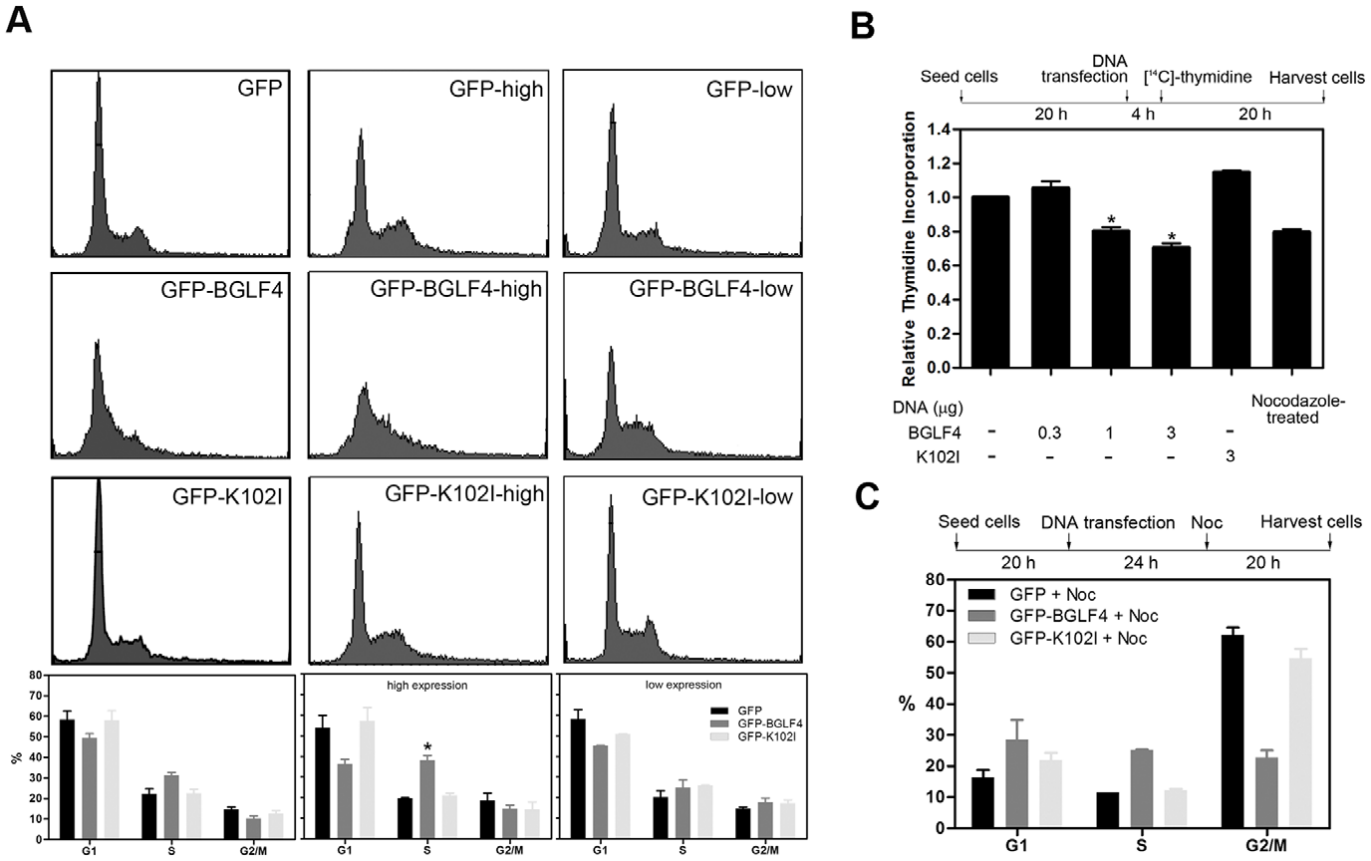
BGLF4 induces the accumulation of the S-phase cell population and represses [^14^C]-thymidine incorporation into HeLa cells. (A) Asynchronous HeLa cells were seeded and transfected with GFP-BGLF4, GFP-K102I expressing plasmids or control GFP vector. At 24 h post transfection, cells were harvested and stained with propidium iodide. The cell cycle profile was analyzed by FACS according to the GFP intensities (left panels). Cells with high GFP expression (middle panels) were further subgrouped from GFP low cells (right panels). The statistics of cell cycle profiles of GFP expressing cells were plotted. Cells with high GFP-BGLF4 expression showed significant accumulation in S-phase (middle panel). An asterisk (*) indicates a statistically significant difference from vector control cells (P<0.05). Two independent experiments were performed and one of the results is shown. (B) At 4 h post transfection, HeLa cells were labeled with [^14^C]-thymidine for newly synthesized DNA. At 20 h post transfection, [^14^C]-thymidine was measured using a liquid scintillation counter. An asterisk (*) indicates a statistically significant difference from vector control cells (P<0.05). Reproducible data were observed in at least three independent experiments. (C) The M-phase synchronous protocol for GFP-BGLF4 or GFP-K102I expressing cells is indicated. HeLa cells were seeded and transfected with GFP-BGLF4, GFP-K102I or GFP vector expressing plasmids. At 24 h post transfection, cells were treated with 200 ng/ml Nocodazole (Noc). After 20 h of Noc treatment, cells were harvested and stained with propidium iodide. Cell cycle profiles of whole cell populations were analyzed by FACS. Two independent experiments were performed.

The DNA content of cells with BGLF4 expression was first analyzed in HeLa cells transiently transfected with GFP, GFP-BGLF4 or GFP-K102I (kinase dead mutant) plasmid. To ensure the levels of ectopically expressed BGLF4 in HeLa cells were comparable to cells replicating the virus, we compared the expression levels in BGLF4-expressing HeLa cells to those in various reactivated EBV positive cell lines, including NA, which is an EBV converted NPC-TW01 cell line [18], EVER8, which is a cell line of 293 background with tetracycline inducible Rta and Akata strain EBV [13], 293-Maxi, which is a 293 derivative cell line harboring the EBV bacmid Maxi [19] and B22, which is a tetracycline inducible BGLF4 expressing cell line. BGLF4 is expressed at a similar level in these cell lines (Figure S1). Then GFP-positive cells were selected by FACS. Consistent with our previous studies, the S-phase cell population of gated GFP-BGLF4-expressing cells increased by approximately 10% (Figure 1A, left panels) [12]. We wondered whether differential expression levels of BGLF4 may lead to different extents of chromosome condensation. Therefore, the DNA contents of cells with various BGLF4 expression levels were analyzed by FACS according to the intensity of GFP fluorescence. Cells with high GFP-BGLF4 expression levels had a salient increase (about 20%) of S-phase DNA content, compared to those cells expressing GFP or GFP-K102I (Figure 1A, middle panels). On the contrary, the S-phase population of cells with low BGLF4 expression levels was not obviously different from those expressing GFP or GFP-K102I (Figure 1A, right panels), suggesting that cells with high BGLF4 expression levels accumulate in S-phase, while cells can proliferate with moderate BGLF4 expression.

### BGLF4 Interferes with Nascent Cellular DNA Synthesis and Cell Cycle Progression

About 40% of cells with high BGLF4 expression levels accumulate at S-phase (Figure 1A), which seems to be conflict with the fact that BGLF4-mediated suppression of MCM4-MCM6-MCM7 DNA helicase activity inhibits cellular DNA replication. To monitor the effect of BGLF4 on DNA synthesis, HeLa cells transfected with BGLF4 or K102I or vector plasmids were subjected to a thymidine incorporation assay to measure nascent DNA synthesis during 4 to 24 h post transfection (Figure 1B). There was a BGLF4 dose-dependent decrease of [^14^C] thymidine incorporation in transfected cells, suggesting that cellular DNA replication was repressed by BGLF4 expression, especially cells with high BGLF4 expression.

To elucidate whether BGLF4 blocks or only delays the S-phase progression, we checked the number of cells that can enter G2/M. To this end, HeLa cells expressing GFP, GFP-BGLF4 or GFP-K102I were treated with the microtubule assembly interfering agent nocodazole (Noc) for 20 h at 24 h post transfection to arrest cell cycle progression at the G2/M boundary (Figure 1C). Total cells with protein expression as indicated were subjected to FACS analysis. More than 50% of HeLa cells with GFP or GFP-K102I expression reached 4 N DNA content after nocodazole treatment at 44 h post transfection. On the contrary, most of the GFP-BGLF4-expressing cells accumulated in G1 or S phase, indicating that these cells cannot bypass G1/S transition or complete S-phase to reach the G2/M boundary in the presence of BGLF4 expression. Thus, the increase of the cell population with DNA contents between 2N and 4N in Figure 1A could be an outcome of the BGLF4-induced repressive effect on DNA synthesis. The increased G1 population observed in Figure 1C may be due to the additional 20 h of BGLF4 expression, suggesting BGLF4 also interferes with G1/S transition. We suggest the suppression of G1/S transition could be due to the blocking of MCM-helicase activity, which is required for the initiation of DNA replication, whereas the retardation of S phase progression may be caused by BGLF4-induced premature chromosome condensation.

### BGLF4 Cannot Compensate for Cdk Activity for S Phase Progression in Temperature-sensitive Mutant Yeasts

**Figure 2:**
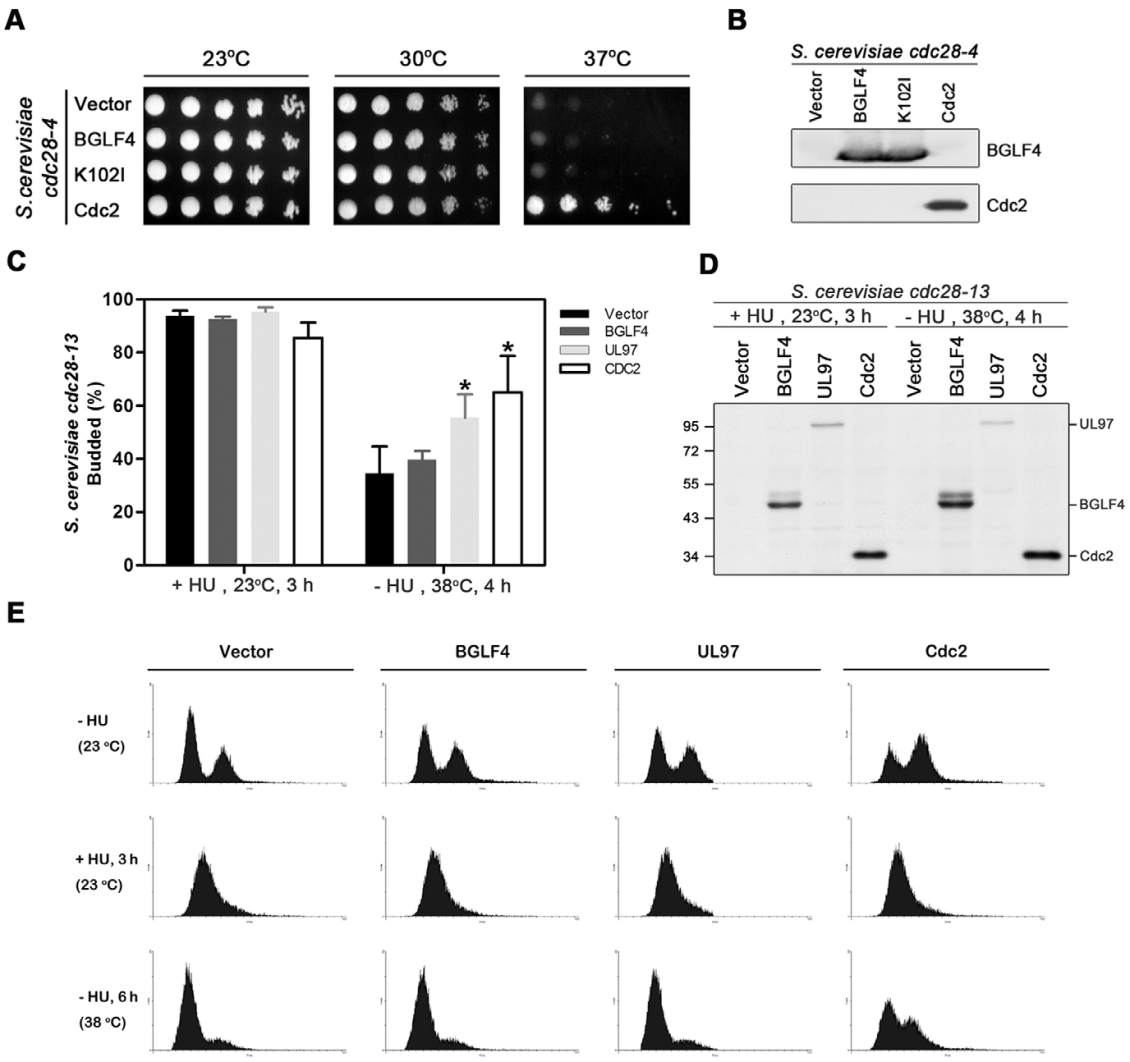
BGLF4 cannot compensate for the kinase activity of Cdk1 for DNA replication in *S. cerevisiae.* (A) Five-fold serial dilutions of *cdc28-4* mutant yeasts containing pVT-U101 vector, BGLF4, K102I, or HA-Cdc2 expressing plasmids were spotted onto YPD plates and incubated at 23°C, 30°C or 37°C for 2 days to observe colony growth. (B) The protein expression levels of BGLF4, K102I and Cdc2 in (A) were detected by immunoblotting. The blot was detected with anti-BGLF4 and anti-HA antibody. (C) Saturated *cdc28-13* cells carrying indicated plasmids were treated with 0.2 M hydroxyurea (HU) at 23°C for 3 h to synchronize transformed yeast cells at an early stage of S-phase. The synchronized cells were released into fresh YPD medium and incubated at the restrictive temperature for 4 h. The percentages of budded cells were determined at the time of temperature shift and 4 h later. The asterisk (*) indicates a statistically significant difference from vector control cells (P<0.01). (D) Total cell extracts of *cdc28-13* transformed with pVTU vector, HA-BGLF4, HA-UL97 or HA-Cdc2 were harvested before and after HU release and subjected to immunoblotting with HA antibody. (E) Asynchro, synchronized or released *cdc28-13* cells containing the plasmids indicated were fixed and stained with propidium iodide to analyze DNA contents by FACS.

Both BGLF4 and its homolog HCMV UL97 have been reported recently to promote the budding of the temperature-sensitive (ts) yeast *cdc28-13* under asynchronous culture conditions at the non-permissive temperature [15]. To determine whether BGLF4 displays S-phase like Cdk activity in yeast, we monitored the function of BGLF4 in the yeast system. The yeast cell cycle is controlled by a single Cdk with various cyclin partners. Yeast Cdk promotes bud emergence, spindle pole body duplication, DNA replication, spindle formation and cell division [20]. Its homologues in prokaryotic and mammalian cells have been identified and proved to compensate the kinase activity in the ts mutant yeast strain *cdc28-4. Cdc28-4* is arrested at G2/M phase and shows budded morphology at the non-permissive temperature [21]. The other Cdk1 ts strain, *cdc28-13*, is appropriate for a budding assay because it is arrested at G1 in a non-budded form at the non-permissive temperature [21]. To determine further whether BGLF4 can compensate for the Cdk defect in *S. cerevisiae cdc28-4*, human Cdc2, BGLF4- or K102I-transformed *cdc28-4* were spotted onto YPD plates and cultured at the permissive (23 or 30°C) or non-permissive (38°C) temperature for 2 days. In contrast to Cdc2-expressing yeasts, BGLF4- and K102I- expressing yeasts did not grow at the non-permissive temperature, indicating that BGLF4 could not provide the Cdc28 activity for cell cycle progression in budding yeasts (Figure 2A and 2B). Similarly, UL97 could not compensate yeast Cdc28 activity in the compensation assay (data not shown). Therefore we wondered whether BGLF4 and UL97 may exhibit only partial Cdk1 activity to promote the advances of bud emergence, as reported, but not DNA replication in yeast. Indeed, bud growth is not absolutely coordinated with the initiation of DNA replication [22]. To determine more precisely whether BGLF4 can promote G1/S transition, we carried out the yeast budding assay in *cdc28-13* cells treated with hydroxyurea (HU), which can block the DNA synthesis, ensuring that most of the cells are in G1 phase. Compared to Cdc2- or UL97-expressing yeast cells, bud growth of BGLF4-transformed yeast cells did not burgeon after the release of non-permissive temperature (38°C) (Figure 2C and 2D). We then analyzed the DNA content of *cdc28-13* cells with different kinases by FACS after HU treatment (Figure 2E). After the treatment, over 80% of *cdc28-13* cells were in a non-budded form and arrested at the G1/S boundary. Although HA-UL97 was immunoprecipitated by HA antibody less effectively, leading to the kinase activity of UL97 being relatively weaker in the IP-kinase reaction (Figure S2), the expression levels of HA-BGLF4, HA-UL97 and HA-Cdc2 were similar in *cdc28-13* cells (Figure 2D). Taken together, the data imply that BGLF4 has kinase activity in yeast but cannot compensate for cdc28 activity for cell growth, promoting yeast budding and S-phase progression. Considering the previous study [15], we believe that the result here is more conclusive because the yeast cells were synchronized before the analysis. The data also are consistent with our observation that BGLF4 activity resembles Cdk-1 activity rather than Cdk-2 activity in mammalian cells.

### Expression Level of BGLF4 is the Key Factor to Determine Cell Fates

**Figure 3:**
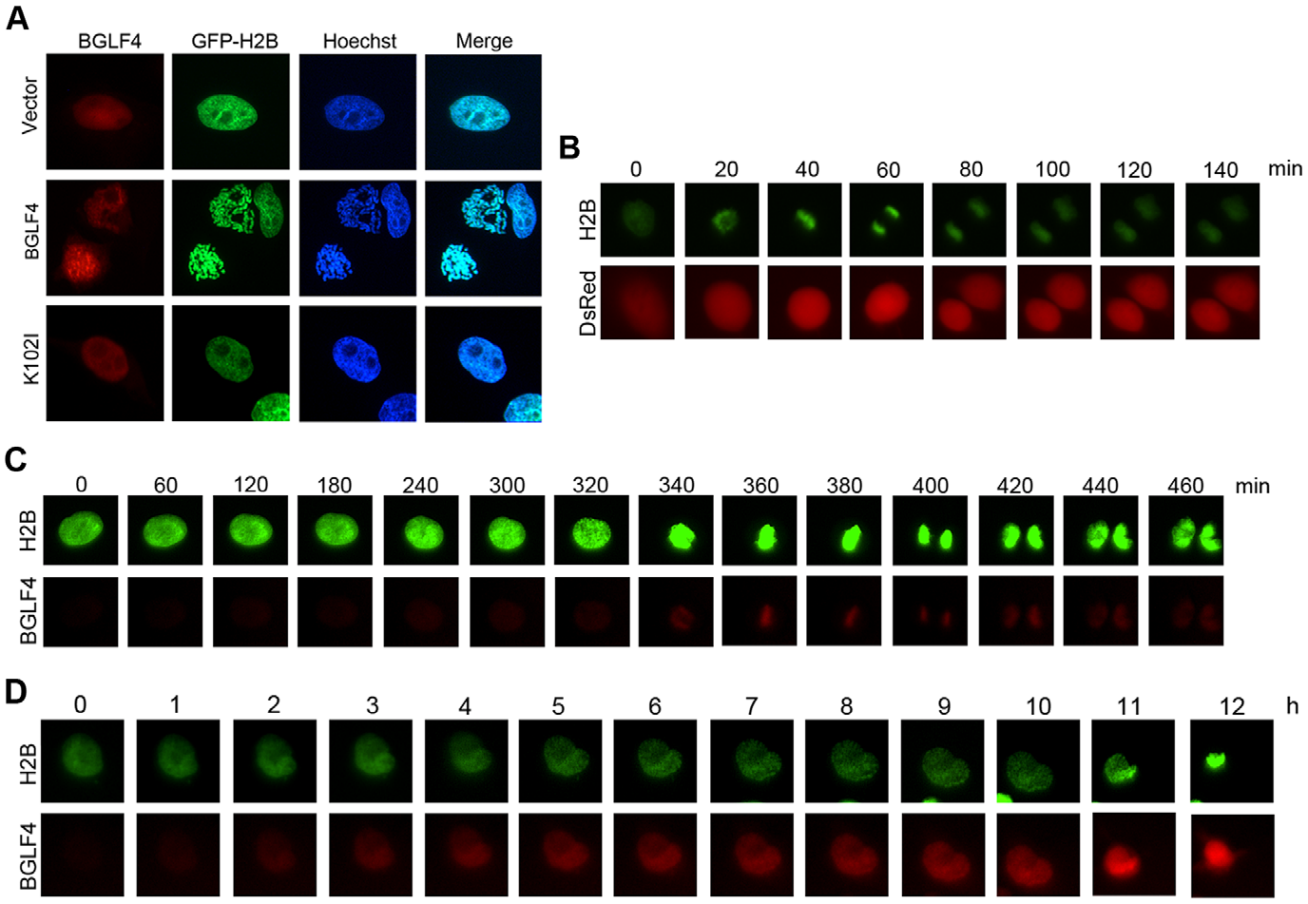
The BGLF4 expression level is the key factor that determines cell fates. (A) Slide-cultured GFP-H2B-HeLa cells were transfected with DsRed-BGLF4, DsRed-K102I and DsRed-monomer. At 24 h post transfection, cells were fixed with 4% paraformaldehyde, stained for DNA using Hoechst 33258 and examined using fluorescence microscopy. (B)(C)(D) GFP-H2B-HeLa cells were seeded and transfected with (B) DsRed-monomer vector or (C)(D) Ds-Red-BGLF4. At 6 h post transfection, fields of view were selected randomly and filmed using an Axiovert 200 M inverted fluorescence microscope every 10 min.

To elucidate the perturbation of cell cycle progression by BGLF4 in the individual cell, we established a GFP-H2B-HeLa cell line stably expressing GFP-H2B to monitor the BGLF4-induced events in living cells. H2B is a component of core histone octamers, which are packaged around the double stranded DNA into the basic subunits of eukaryotic chromosomes. The chromosomes of living cells can be visualized through GFP-H2B observation with fluorescence microscopy [23]. We found DsRed-BGLF4, but not DsRed-K102I or DsRed, induced chromosome condensation in GFP-H2B-HeLa cells at 24 h post transfection. In addition, GFP-H2B signals colocalized with the Hoechst 33258-stained DNA, suggesting that this is a good indicator of the chromosome pattern in living cells (Figure 3A).

To identify the fate of individual cells with BGLF4 expression, DsRed vector control or DsRed-BGLF4 expressing plasmid was transfected into GFP-H2B-HeLa cells. Six hours post transfection, fields of view were selected randomly and imaged using time-lapsed fluorescence microscopy for 24 h to determine whether the cells could grow and divide normally after recombinant protein expression. As expected, cells transfected with DsRed vector could complete mitosis (Figure 3B). On the contrary, chromosomes in cells with high BGLF4 expression levels condensed severely, aggregated and eventually became puckered (Figure 3D), while chromosomes in cells with low BGLF4 expression showed a punctuate or loose condensation pattern and could segregate into two daughter cells (Figure 3C). Only cells expressing low levels of BGLF4 completed cytokinesis (32 out of 103 BGLF4 expressing cells). According to the appearance of condensed chromosomes up to the separation of two daughter cells, the cells with low BGLF4 expression spent a longer period of time in mitosis (about 80 min) than control cells (about 60 min) while completing cytokinesis, suggesting the G2/M checkpoint could be activated. Taken together, we propose that BGLF4 expression levels are critical in determining cell fates.

### Expression of BGLF4 in 293 T-REx and TW01 T-REx BGLF4 Inducible Cells Leads to Growth Retardation

To observe the long-term effects of BGLF4 on cell growth, BGLF4 inducible cells were established in a 293 tetracycline-regulated expression system (293 T-REx). In the following studies, clone V4 carrying vector, clones B9, B10, B17, B19, B20 and B22 carrying BGLF4, or clone K9 carrying K102I were selected to determine their protein expression levels as detected by immunoblotting and indirect immunofluorescence assays (Figure S3A and S3C). BGLF4 could be detected at 3 h post induction (hpi) and persisted for at least 120 hpi in B22 cells (Figure S3B). The premature chromosome condensation phenomenon induced by BGLF4 was observed at 6 hpi, accompanied by its nuclear distribution (Figure S3D), indicating that BGLF4 is biologically functional in this model. BGLF4 expression levels in B22 cells were also compared to reactivated EBV positive cells to verify comparable levels of BGLF4 expression in this system (Figure S1).

**Figure 4:**
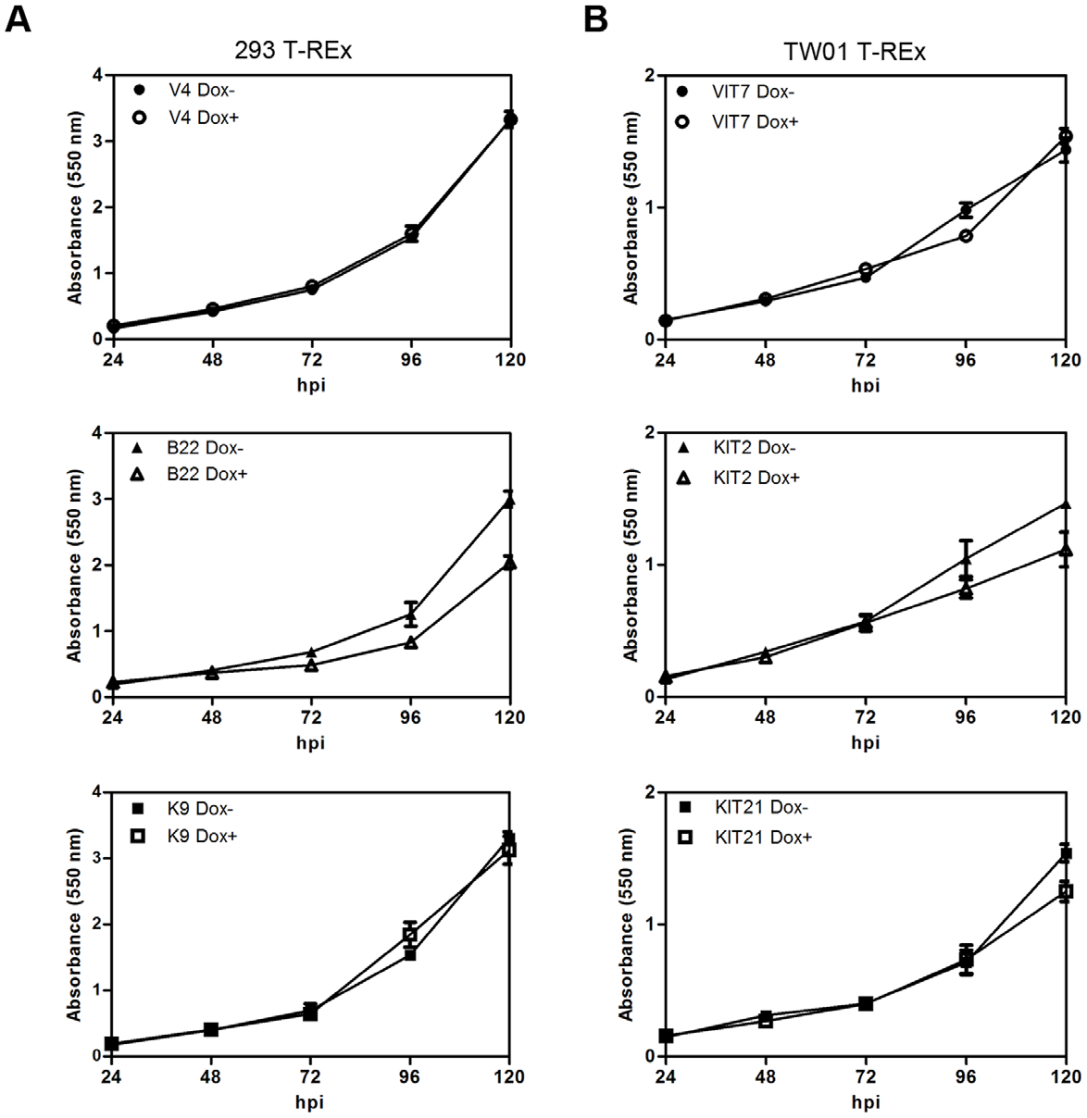
Expression of BGLF4 results in slight cell growth retardation. (A) 293 T-REx BGLF4 inducible B22, K102I kinase dead K9 and vector control V4 cells were seeded into 96-well plates in triplicate and incubated in 100 ng/ml Dox containing medium to induce the expression of BGLF4 or K102I. At 24, 48, 72, 96 and 120 h post induction (hpi), MTT assays were performed and the optical densities (OD) were determined by spectrophotometry at 550 nm. Three independent experiments were conducted and one representative result is shown. (B) NPC-TW01 T-REx BGLF4 inducible KIT2, KIT21 cells and vector control VIT7 cells were induced with 50 ng/ml Dox and subjected to an MTT assay as described in (A).

To determine whether BGLF4 affects cell proliferation, V4, B22 and K9 cells were induced with doxycycline (Dox, 100 ng/ml) and assessed for cell proliferation by MTT assay every 24 h until 120 hpi. Compared to V4 and K9 cells, B22 cells exhibited cell growth retardation following Dox induction (Figure 4A). At 120 hpi, the percentage of cell proliferation reduced to about 30% (P<0.01) in B22 cells (the conversion equation is described in materials and methods). This phenomenon also could be observed in the other 293 T-REx BGLF4 inducible cell lines, B9 and B20, after Dox induction (Figure S3E). In the case of these two cell lines, the cell proliferation was reduced to about 30% and 35%, respectively. To explore further whether BGLF4 can affect the growth of NPC cells, the tetracycline-regulated expression system also was established in NPC-TW01 cells. VIT7 carrying vector, KIT2 and KIT21 carrying BGLF4 were selected according to the protein expression levels detected by immunoblotting and immunofluorescence assays (Figure S4A and S4C). Similar to B22, BGLF4 could be detected at 3 h post induction, and persisted for at least 120 hpi in KIT2 (Figure S4B). Also, the cell growth was examined in the NPC-TW01 cell background. Both KIT2 and KIT21 grew slower than VIT7 cells (Figure 4B). The cell proliferation rate decreased to about 24% and 19% respectively. Nevertheless, in both the 293 and TW01 cell background, cells expressing BGLF4 can still grow continuously. Considering the effect of BGLF4 on inducing prophase-like chromosome condensation, it is possible that some cells may proliferate with damaged DNA.

### BGLF4 Elicits Genomic Instability in 293 and NPC-TW01 Cells

**Figure 5:**
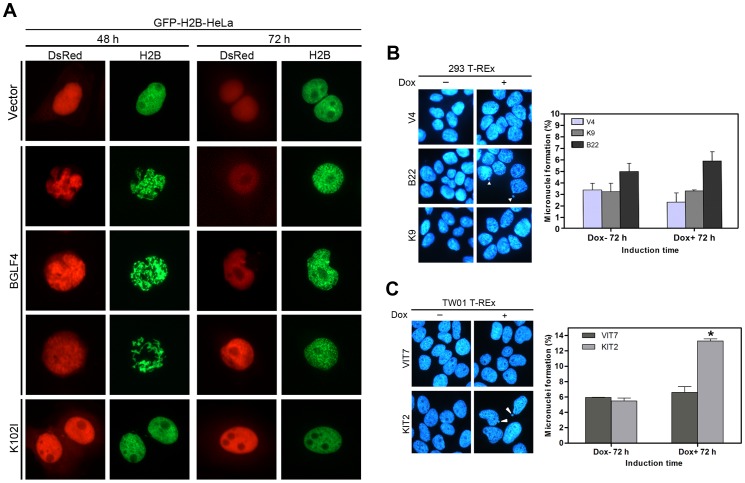
Expression of BGLF4 elicits abnormal chromosomal structure and micronuclei formation in various cell types. (A) Slide-cultured HeLa cells with stable GFP-H2B expression were transfected with DsRed-BGLF4, DsRed-K102I or pDsRed-monomer. At 48 h or 72 h post transfection, cells were fixed, stained for DNA with Hoechst 33258 and examined using fluorescence microscopy. (B) Slide-cultured 293 T-REx B22, K9, and V4 cells were treated with 100 ng/ml doxycycline (Dox). At 72 h post induction (hpi), cells were fixed with 4% paraformaldehyde and stained for DNA with Hoechst 33258. Micronucleus (MN) formation was observed using fluorescence microscopy. Detection of MN formation (MN%) was calculated as the percentage of cells containing micronuclei, derived from the analysis of 1,000 cells. Bars represent mean values in MN% (duplicates±SD). One of the two independent experiments conducted is shown. (C) Slide-cultured NPC-TW01 T-REx KIT2 and VIT7 cells were incubated with 50 ng/ml Dox for 72 h. Cells were fixed with 4% paraformaldehyde and examined using fluorescence microscopy. Detection of MN formation (MN%) was calculated as the percentage of cells containing micronuclei, derived from the analysis of 1,200-1,600 cells. An asterisk (*) indicates a statistically significant difference from vector control cells (P<0.01). One of the two independent experiments conducted is shown.

Micronuclei (MN) represent unincorporated chromosomal fragments lost during sister chromatid segregation in mitosis and are a common indicator for evaluating genomic instability [24]. Repetitive reactivations of EBV have been considered recently to promote MN formation and tumor progression in NPC cells [6]. In addition, premature chromosome condensation induced by calyculin A, which is a Ser/Thr phosphatase types 1 and 2A inhibitor, was found prone to chromosomal breaks at common fragile sites [25]. Therefore, we suggest BGLF4-expressing cells may proliferate with damaged DNA and genomic instability. To this end, we observed the chromosome pattern of DsRed-BGLF4-transfected GFP-H2B-HeLa cells at 48 h post transfection and found the formation of framework-shaped chromosome structure (Figure 5A), implying that cells suffering BGLF4-induced chromosome condensation might undergo further chromosomal alterations. Since we did not observe similar patterns at 72 h post transfection, it is possible that those cells with dramatic chromosome abnormalities may die eventually.

Subsequently, micronucleus (MN) formation was examined in 293 T-REx B22, K9 and V4 cells after Dox induction for 72 h. After induction, BGLF4 enhanced MN formation in 293 T-REx B22 (Figure 5B), implying that persistent expression of BGLF4 elicits genomic instability. Compared to vector control cells and K9 cells, the MN formation also was increased in non-induced B22 cells. We suspected that there might be a very small amount of BGLF4 expressed in B22 cells before induction (Figure S3A), leading to the increase of MN. Notably, the MN occurrence in BGLF4 expressing NPC-TW01 cells (13.03%) was much more obvious than that in control cells (5.97%, P<0.01) (Figure 5C). In addition to MN, donut-shaped, new moon-shaped, horse’s hoof-shaped and other irregular chromosome patterns (37.08%) were observed in KIT2 cells after Dox induction for 72 h, while the percentage of abnormal chromosomes observed in VIT7 cells was only one-third (13.99%) of that in KIT2 cells (data not shown). We examined two other BGLF4 inducible clones (KIT20 and KIT21). MN formation and abnormal chromosomal structure also can be observed at 60 hpi (data not shown). Taken together, these results suggest that BGLF4 elicits genomic instability more severely in NPC cells than in 293 cells.

### The G1/S Transition Regulator RB is Hyperphosphorylated but the RB-E2F1 Complex is not Disrupted in the Presence of BGLF4

Our data above indicate that BGLF4 does not promote S-phase progression. We noticed that in a previous study, only RB phosphorylation, but not the downstream E2F1 transactivation activity, was monitored in the presence of BGLF4 [15]. Therefore, we decided to clarify the RB phosphorylation pattern and its downstream signaling in cells expressing BGLF4. RB is known for its essential role in sequestering E2F1through a distinct pocket domain, resulting in inhibition of E2F transcription activity [26], [27]. RB is sequentially phosphorylated by cyclin-Cdks in a cell cycle dependent manner [28], [29]. The hyperphosphorylated form of RB dissociates the RB-E2F1 complex, allowing E2F1 downstream genes required for DNA synthesis and G1/S transition to be transcribed [30]. There are 16 Cdk-consensus sites within RB. Ser780 has been shown to be a critical residue for the subsequent hyperphosphorylation of multiple sites at the C-terminus and disruption the RB-E2F1 complex [31], [32]. Ser608 and Ser612 within the spacer domain are also required for the disruption of the interaction at G1/S transition [32].

**Figure 6:**
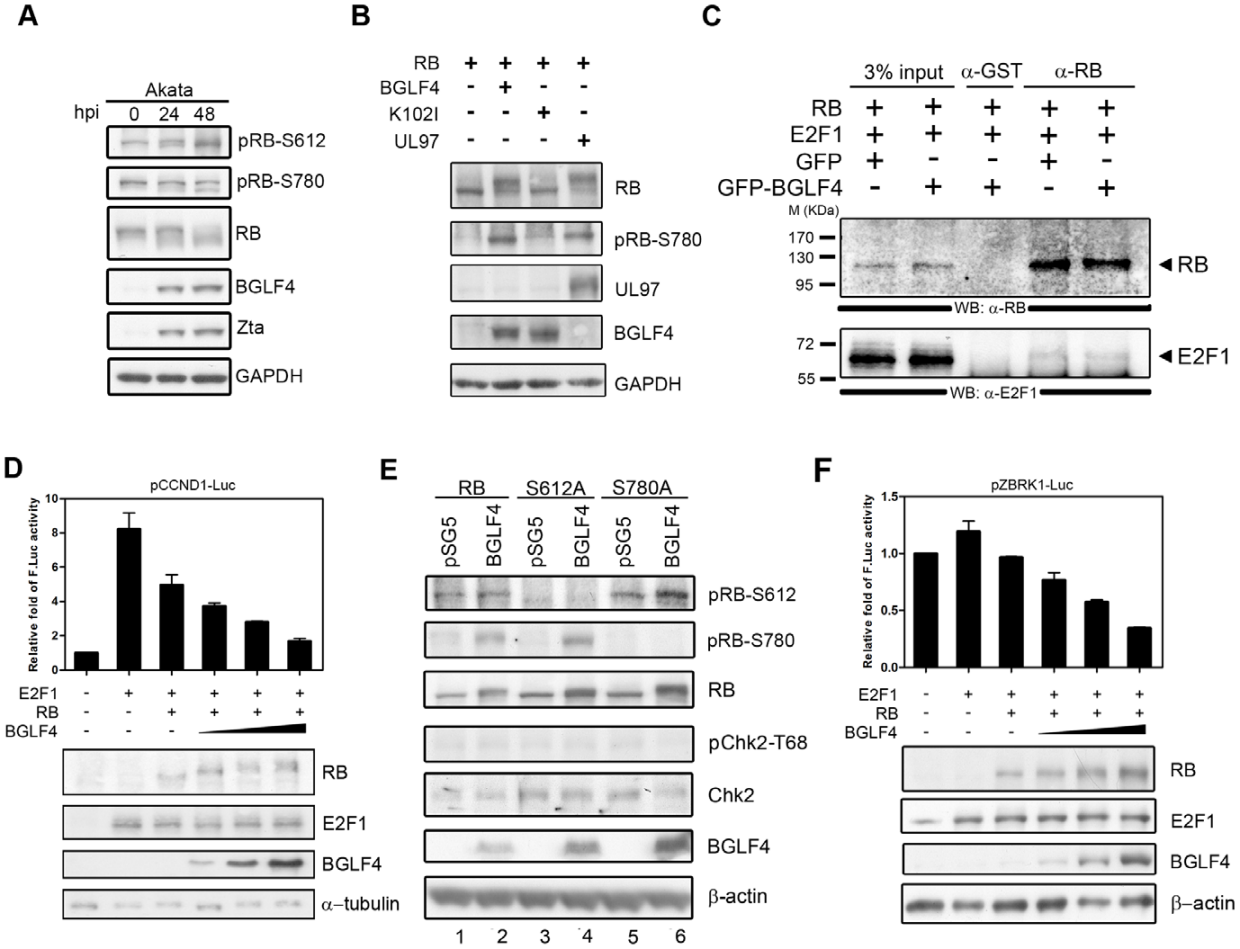
RB is hyperphosphorylated but the RB-E2F1 complex is not disrupted in the presence of BGLF4. (A) EBV-positive Akata cells were induced by 0.8% anti-human IgG and harvested at the time points indicated. The proteins were resolved by 7.5% SDS-PAGE (for total RB) or 10% SDS-PAGE (for RB specific phosphorylation, Zta, BGLF4 and GAPDH). Zta and BGLF4 were detected to confirm lytic cycle progression. GAPDH served as a loading control. (B) Saos-2 cells were transfected with the expressing plasmids indicated. Cells were harvested at 24 h post transfection. The protein expression levels were resolved by 7.5% SDS-PAGE (for RB) or 10% SDS-PAGE (for BGLF4, HCMV UL97, RB specific phosphorylation and GAPDH). GAPDH served as a loading control. (C) Saos-2 cells were transfected with the plasmids indicated. Cells were harvested at 24 h post transfection and cell extracts were subjected to immunoprecipitation assays using anti-RB antibody. The immunocomplexes were separated by 10% SDS-PAGE. The co-immunoprecipitated proteins RB and E2F1 were detected using specific antibodies. (D) Luciferase reporter assays were performed in Saos-2 cells by co-transfected with p-CCND1-luc (a 3.3-kb full length cyclin D1 promoter construct) as well as pWP1 (for normalization of transfection efficiency) and plasmids of effectors as indicated. Luciferase activities were measured at 48 h post transfection. Results are means ± SD from three separate transfections. Data are representative of two independent experiments. (E) Saos-2 cells were transfected with the expression plasmids as indicated. Cells were harvested at 48 h post transfection. The protein expression levels were resolved by 7.5% SDS-PAGE (for RB) or 10% SDS-PAGE (for BGLF4, Chk2, RB specific phosphorylation and β-actin). β-actin served as a loading control. (F) Luciferase reporter assays were performed in Saos-2 cells by co-transfection of p-ZBRK1-luc (containing ZBRK1 promoter fragment -624 to +47 bp) as well as pWP1 (for normalization of transfection efficiency), and plasmids of effectors as indicated in the figure. Luciferase activities were measured at 48 h post transfection. Results are means ± SD from two separate transfections. Data are representative of two independent experiments.

Nevertheless, RB is alternatively phosphorylated with different patterns under different signaling situations. Ser612 is phosphorylated by Chk1/2 in response to DNA damage, leading to a complex stabilization between RB and E2F1 [33]. As a result, phosphorylation of RB at different sites by various kinases may result in different functions. Hyperphosphorylated forms of RB were observed in EBV-positive cells. Both the phosphorylation on Ser612 and Ser780 increased after EBV reactivation [4]. In addition, RB was shown to be hyperphosphorylated in response to the ectopic expression of BGLF4 and other CHPKs, including HCMV UL97 [15].We wondered the BGLF4-induced RB hyperphosphorylation could be a response to DNA damage singling but not for DNA replication. To this end, we first examined the RB phosphorylation pattern in EBV-positive Akata (Figure 6A) and NA cells (data not shown) upon reactivation. Consistent with the previous observation [4], the increase of phosphorylation of total RB and Ser780 was not so obvious, while phosphorylation on Ser612 was elevated during lytic virus replication, accompanied by the expression of BGLF4 in Akata cells (Figure 6A).

To investigate the effects of BGLF4 on hyperphosphorylation of RB, RB-deficient Saos-2 cells were transfected with plasmids expressing BGLF4, K102I, or vector along with an RB expressing plasmid. HMCV UL97 was added to the experiment as a comparison for RB hyperphosphorylation [15], [34]. In contrast to K102I or vector transfected cells, cells expressing BGLF4, as well as UL97, showed a band-shift hyperphosphorylated RB (Figure 6B) and increased phosphorylation on Ser780, as reported previously [15]. To elucidate the effect of RB phosphorylation on the stability of the RB-E2F1 complex, an immunoprecipitation assay was performed with anti-RB antibody and the immunocomplexes were detected with anti-RB and anti-E2F1 antibodies. Throughout several repeated experiments, weak but consistent results were observed that the amount of E2F1 co-precipitated with anti-RB antibody in the presence of BGLF4 expression was not obviously different (Figure 6C). We assumed the RB phosphorylation pattern observed here does not contribute to cell cycle progression. To support this hypothesis, the promoter activity of the E2F1 downstream gene cyclin D1 (p-CCND-luc, which contains a 3.3 kb promoter region of cyclin D1) was examined in a reporter assay (Figure 6D). Cyclin D1 promoter activity was enhanced by E2F1 alone and suppressed when RB was co-transfected with E2F1. Upon ectopic BGLF4 expression, the RB-inhibited cyclin D1 promoter activity was suppressed further. Furthermore, BGLF4 had a dose-dependent effect on the down-regulation of cyclin D1 promoter activity and the hyperphosphorylation of RB (Figure 6D), suggesting that expression of BGLF4 does not favor cell cycle progression. This observation implies that, in addition to Ser780, at which phosphorylation is required for E2F1 dissociation from RB, BGLF4 may also induce phosphorylation at other residues, to affect RB-downstream signaling.

We then elucidated whether BGLF4-elicited RB phosphorylation is a downstream effect of DDR. We generated RB Ser612 and Ser780 phosphorylation mutants and used phospho-specific antibodies to determine whether BGLF4 enhances the phosphorylation on RB Ser612 (Figure 6E). Although the phosphorylation on Ser780 was more obvious than that on Ser612 of wild-type RB (lanes 1 and 2), we noticed that the BGLF4-induced RB band shift was reduced in S612A but not in S780A (lanes 3 and 4, lanes 5 and 6), implying that Ser612 may be a major target of BGLF4. It is worthy of note that BGLF4 also led to RB phosphorylation on Ser612, even though Chk2 was not obviously activated under this condition, suggesting that BGLF4 has the potential to phosphorylate RB directly at Ser612. We then examined the promoter activity of ZBRK1, a transcription factor negatively regulated by the RB-E2F1 complex at the transcription level upon DDR [35]. ZBRK1 promoter activity was slightly down-regulated when RB and E2F1 were co-expressed in Saos-2 cells (Figure 6F). In the presence of BGLF4, the ZBRK1 promoter activity was suppressed further in a dose dependent manner (Figure 6F), implying that BGLF4-elicited RB hyperphosphorylation is in response to DNA damage signaling but not for cell cycle progression.

## Discussion

BGLF4 is a member of the conserved herpes protein kinases (CHPKs), which are known to mimic cellular Cdk activities but are not restrained by Cdk inhibitors [10], [12]. BGLF4 induces multiple mitotic-like events [12], [13], induces RB hyperphosphorylation [15] and elicits DDR [17] in a cell cycle-independent manner. These changes triggered by BGLF4 appear to be contradictory in terms of cell cycle control. Here we have shown BGLF4 interferes with DNA replication and S-phase progression, restraining cells from entering G2/M phase. Observation of individual BGLF4 expressing cells using time-lapse microscopy supports this finding. Once BGLF4 induces highly condensed chromosomal structures, cells cannot complete the cell cycle. Nevertheless, cells with moderate BGLF4 expression still went through cytokinesis with a time delay. To avoid the influence of endogenous Cdk activity, we used a temperature-sensitive yeast system to determine whether BGLF4 promotes S-phase progression. BGLF4 could not promote bud growth or S-phase progression of the *cdc28-13* strain synchronized at the G1 phase. These findings suggest that BGLF4 behaves more like Cdk1 than Cdk2. On investigating the G1/S transition regulator, RB, we found that, although RB was hyperphosphorylated in the presence of BGLF4, the RB-E2F1 complex did not dissociate. The reporter assays further demonstrate that BGLF4 induced a dose-dependent suppression of E2F1 downstream cyclin D1 and ZBRK1 promoter activities.

Recently, it was shown in the fission yeast model that the cell cycle can be driven by oscillation of a minimal Cdk control network lacking canonical regulation such as CAK (Cdk activating kinase) and Cdk inhibitors [36]. It was proposed that the Cdk oscillator acts as the primary organizer of the cell cycle by setting two thresholds of Cdk activity to define independent cell cycle phases. Notably, G1/S and G2/M transitions are associated with low and high Cdk activities, respectively. This model supports our data regarding BGLF4 mediated S-phase disturbance, because BGLF4 resembles constitutive Cdk activities throughout the cell cycle.

**Figure 7:**
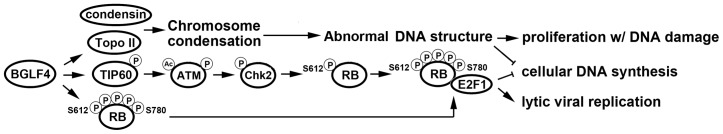
A hypothetical model of BGLF4-induced RB phosphorylation and DNA damage response (DDR). BGLF4 induces premature chromosome condensation through activation of condensin and Topo II. The prophase-like chromosomes may cause abnormal DNA structure and micronucleus formation. In addition, BGLF4 elicits DNA damage signaling by phosphorylating TIP60. TIP60 is a histone acetyltransferase that regulates chromatin remodeling and the DNA damage response (DDR). Upon DNA double-stranded breakage, ATM kinase is acetylated by TIP60. The acetylation of ATM is essential for its autophosphorylation and activation. Therefore, BGLF4 is able to turn on DNA damage signaling in a TIP60/Chk2-dependent manner. Here we found that the exogenous expression of BGLF4 induces RB hyperphosphorylation at Ser780 and Ser612, which is an indicator of DNA damage signaling activation. Based on our observation that the RB-E2F1 complex does not dissociate and the E2F1-downstream cyclin D1 promoter and ZBRK1 activities are repressed upon BGLF4 expression, we propose that BGLF4-induced RB phosphorylation may be a downstream event of DDR. Because the DNA damage pathway is necessary for efficient EBV replication, BGLF4-induced RB phosphorylation is favorable for lytic cycle progression, while cellular DNA synthesis is blocked.

Uncoordinated RB hyperphosphorylation and cell cycle progression upon EBV reactivation also was reported in another study. In this condition, RB was hyperphosphorylated because of the elevated expression of S-phase cyclins and upregulated S-Cdk activity but host DNA replication and cell cycle progression were blocked [4]. It is interesting to note that phosphorylation of RB on Ser612 increased more dramatically than on Ser780, following EBV reactivation. The phosphorylation on Ser780 even decreases slightly at 72 hpi [4]. Here, we also demonstrate similar RB phosphorylation patterns in EBV positive Akata (Figure 6A) and NA (data not shown) cells after lytic cycle reactivation. Overall, a hypothetical model is proposed to illustrate the effects of BGLF4 on RB phosphorylation and cell cycle progression in cells replicating the virus (Figure 7). BGLF4 is expressed with an early-late kinetic pattern, its expression starting at an early stage of replication and increasing after viral DNA replication. We suggest that at the early stage, low concentrations of BGLF4 do not affect the host cell cycle but are sufficient to enhance the transactivation activity of Zta and the synergistic activation of BMRF1 and Zta on the BHLF1 promoter within the lytic origin of replication (oriLyt) [37]. Along with the progression of viral DNA replication, BGLF4 expression reaches the maximal level and induces premature chromosome condensation and arrest of cellular DNA synthesis. A subset of cells may proliferate with abnormal DNA structure. Phosphorylation of RB on Ser612 but not Ser780 was more obvious at a later stage of the lytic cycle, suggesting it as a downstream effect of EBV-elicited DDR in the cells (Figure 6 A). Alternatively, BGLF4 may activate TIP60 histone acetyltransferase and induce the ATM-mediated DDR pathway, leading to the phosphorylation of RB on Ser612 [17]. Because Chk2 was not significantly activated in Saos-2 cells in our experiments, it is very likely that Ser612Pro613 of RB can be phosphorylated directly by BGLF4. This conclusion is supported by the observation that the increase of RB Ser612 phosphorylation at a later stage of the lytic cycle (48 h post induction) is correlated with higher BGLF4 expression. Because the DNA damage pathway is necessary for efficient EBV replication, we proposed that BGLF4-induced RB phosphorylation is favorable for lytic cycle progression, while cellular DNA synthesis is blocked.

Uncontrolled and constitutive Cdk activities may contribute to unscheduled proliferation, as well as genomic instability (GIN) and chromosomal instability (CIN), because switch-off of Cdk1-like activity is required for correct sister chromatid separation, chromosome decondensation, nuclear envelope reformation and cytokinesis [38], [39]. Therefore, deregulation of Cdk1 activity and the consequent deregulated function of Cdk1 substrates may contribute to abnormal genome organization [39]. In this study, we used BGLF4-inducible 293 and NPC-TW01 cells to observe the long-term effects of BGLF4 on chromosome structures. In addition to MN formation, BGLF4 elicits abnormal chromosome structure, particularly in NPC-TW01 cells. Several aspects of BGLF4 function may contribute to genomic instability. In our previous studies, BGLF4 was shown to induce some mitosis-like events, including premature chromosome condensation, through topoisomerase II (Topo II) activation and nuclear lamina disassembly through lamin A/C phosphorylation. Under physiological conditions, these events are tightly controlled in a cell-cycle dependent manner and occur only in M-phase. Topo II is a ubiquitous enzyme regulating DNA topology through a transient double-stranded break. As a result, Topo II functions like a “double-edged sword”, being essential for cells but potentially genotoxic at a high level of protein expression. With abnormally high levels of Topo II, double-stranded breaks may become permanent, leading to chromosomal translocation and other DNA aberrations [40]. Indeed, increased levels of Topo II are associated with mixed lineage leukemia (MLL) [41], [42] and breast cancer [43]. On the other hand, lamin A/C is the component of nuclear lamina, constituting a supporting meshwork to maintain nuclear structure. The integrity of lamina is crucial to nuclear stiffness and the spherical shape of nuclei [44]. BGLF4 phosphorylates lamin A at amino acids 22, 390, 392, 652 and 657, leading to the disassembly and reorganization of the nuclear lamina [13]. As a result, BGLF4 may elicit CIN at the nuclear structure level, too. Furthermore, it is speculated that premature chromosome condensation induces chromosome breaks at common fragile site [25], reinforcing the linkage of BGLF4 and CIN. Thus, BGLF4 may be one of the important factors that mediate recurrent, reactivation-induced genomic instability in EBV positive NPC cells [6]. Here, we showed that BGLF4 may be involved in multiple pathways leading to GIN, suggesting that BGLF4 may play a role in EBV-associated epithelial tumor progression.

## Materials and Methods

### Plasmids

pYPW17 and pYPW20 are pSG5 (Promega) based plasmids which express BGLF4 and K102I, as described previously [45]. To generate the tetracycline regulated expression plasmids pLenti4-BGLF4 and pLenti4-K102I, a DNA fragment of BGLF4 open reading frame 1-1290 with RsrII restriction enzyme sites at both ends was PCR amplified using pYPW17 or pYPW20 as template and forward primer LMRC481 (5′-CACGGTCCGACCATGGATGTGAATATGGCTGC-3′) and reverse LMRC482 (5′-CACGGACCGTCATCCACGTCGGCCATCT-3′). The PCR product was digested with RsrII and subcloned into the RsrII site of pLenti4-TO-V5-His (Invitrogen). pCPL4 and pTHL5 are plasmids expressing GFP-BGLF4 and GFP-K102I, generated by cloning a BamHI-EBV BGLF4-EcoRI fragment from pYPW17 or pYPW20 into the BglII-EcoRI cloning site of pEGFP-c1 (BD Biosciences). pYHC3 and pYHC4 are plasmids expressing DsRed-BGLF4 and DsRed-K102I, generated by cloning the BamHI-BGLF4-BglII fragments from pYPW17 and pYPW20 into the BamHI site of pDsRed-monomer-c1 (Clonetech). CMV-NeoRB, which contains pRB cDNA in pCMV-neo-Bam, was kindly provided by Dr. Phang-Lang Chen (Department of Biological Chemistry, University of California, Irvine, CA) [46]. For RB phosphorylation mutants, RB-S612A and RB-S780A were generated by a single-primer-based site-directed mutagenesis strategy using CMV-NeoRB as template and forward primer LMRC855 (tatgtatctttctcctgtaagagctccaaagaaaaaaggttcaac) or LMRC856 (tccaccaggccccctaccttggcaccaatacctcacattcctc) respectively. pCMV-E2F1 was a gift from Dr. Sheau-Yann Shieh (Institute of Biomedical Sciences, Academia Sinica, Taiwan) [47]. CCND1 reporter plasmids (CCND1-P) containing the 3.3-kb cyclin D1 promoter, and ZBRK1 reporter plasmids (ZBRK1-B) containing ZBRK1 promoter fragment -624 to +47 bp, were a gift from Dr. Ju-Ming Wang (Institute of Basic Medical Sciences, National Cheng Kung University, Taiwan) [35]. pEGFP-H2B, which expresses a GFP-H2B fusion protein, was generously provided by Dr. Zee-Fen Chang (Institute of Biochemistry and Molecular Biology, National Yang-Ming University, Taiwan).

### Cell Culture and Transfection

HeLa cells (ATCC: CCL-2), derived from a human cervical carcinoma, were grown in Dulbecco’s modified Eagle’s medium (DMEM) with 8% fetal calf serum. Saos-2 (ATCC: HTB-85), an RB deficient cell line derived from a human primary osteosarcoma, was grown in DMEM medium with 8% fetal calf serum. 293 T-REx cell line (Invitrogen) is a HEK293 derivative containing a Tet repressor. NPC-TW01 was derived from an EBV-negative human nasopharyngeal carcinoma [48]. Cells were transfected using Lipofectamine 2000 (Invitrogen) in OptiMEM medium (GIBCO-BRL) according to the manufacturer’s instructions. TW01 T-REx cells stably expressing the Tet repressor were generated using the ViraPower T-REx lentiviral expression system (Invitrogen). 293 T-REx and TW01 T-REx cell lines were grown in DMEM medium with 8% tetracycline-free fetal calf serum. To establish tetracycline-regulated BGLF4 inducible cell lines, 293 T-REx or TW01 T-REx cells were transfected with pLenti4, pLenti4-BGLF4 or pLenti4-K102I and selected with zeocin (400 µg/ml for 293 T-REx or 500 µg/ml for TW01 T-REx) and blasticidin (5 µg/ml for 293 T-REx or 25 µg/ml for TW01 T-REx) for two months. The V4 clone carrying vector, B9, B10, B17, B19, B20 and B22 clones carrying the BGLF4 expression plasmid and K9 clone carrying the K102I expression plasmid were selected from 293T-Rex cells. The VIT7 clone carrying vector, as well as KIT2 and KIT21 carrying the BGLF4 expression plasmid, were picked out from TW01 T-REx for further studies. The selected clones were maintained in the presence of zeocin (200 µg/ml for 293 T-REx or 250 µg/ml for TW01 T-REx) and blasticidin (2.5 µg/ml for 293 T-REx or 12.5 µg/ml for TW01 T-REx). To induce protein expression, doxycycline (Dox) was added (100 ng/ml for 293 T-REx or 50 ng/ml for TW01 T-REx) to the culture medium. The inducibility and positive rate of BGLF4 in individual clones were examined by immunoblotting and indirect immunofluorescence assay (IFA) after Dox treatment for 24 h. The induction rates of both cell lines were more than 95%, as revealed by IFA. To establish HeLa cells stably expressing GFP-H2B (GFP-H2B-HeLa), HeLa cells were transfected with a GFP-H2B expression plasmid and selected with G418 (1 mg/ml) for 1 month. The clone 3A1 with more than 95% GFP-H2B positive cells in IFA was used for further experiments. All cells were maintained in the presence of 1 mM L-glutamine and penicillin (100 U/ml) and streptomycin (100 µg/ml) at 37°C with 5% CO_2_. Akata cell line with recombinant EBV [49] that expressed enhanced green fluorescent protein was grown in RPMI 1640 medium with 8% fetal calf serum and reactivated with 0.8% anti-human IgG.

### Cell Cycle Synchronization and Fluorescence-activated Cell Sorter (FACS) Analysis

GFP-BGLF4-transfected HeLa cells were treated with nocodazole (200 ng/ml) for 20 h at 24 h post transfection to arrest them at the G2/M boundary. After synchronization, cells were trypsinized, washed with PBS, fixed with 1% paraformaldehyde at room temperature for 20 min and then with 75% ice-cold ethanol overnight. Before FACS analysis, cells were permeabilized with 0.2% Triton X-100 and RNase A (100 ng/ml) and DNA was stained with propidium iodide (10 µg/ml) at room temperature for 30 min. Finally, cell cycle distribution according to DNA contents was determined using a FACSCalibur cell sorter and analyzed by CellQuest software (BD Biosciences).

### Thymidine Incorporation Assay

HeLa cells were seeded in 6-well plates (3×10^5^/well) and transfected with BGLF4, K102I expressing plasmid or vector. At 4 h post transfection, cells were labeled with [^14^C]-thymidine (0.05 µCi/ml) for 20 h. Subsequently, cells were trypsinized, washed with PBS and lysed in 0.8 ml 0.2N NaOH. Half of the NaOH lysates was added to 4 ml Ready-safe liquid scintillation liquid (Beckman Coulter) and counted in a liquid scintillation analyzer (LS 6000 TA, Beckman Coulter).

### Immunoblotting and Co-immunoprecipitation

Cell extracts harvested with NP-40 lysis buffer (1% NP-40, 50 mM Tris, pH 8.0, 150 mM NaCl, 2 mM EDTA, and 1 mM Na_3_VO_4_) were resolved by SDS-polyacrylamide gel electrophoresis (7.5% acrylamide gel for RB detection or 10% acrylamide gel for the detection of other proteins). After transfer and blocking, the primary antibodies used were Zta mAb (1B4) [50], BGLF4 mAb (2616) [45], GAPDH mAb (Biodesign), α-tubulin mAb (Calbiochem), β-actin mAb (AC-15, Sigma), RB mAb (pMG3-245, kindly provided by Dr. Wen-Hwa Lee (Department of Biological Chemistry, University of California, Irvine, CA)), phosphoPlus-RB Ab Kit (#9969, Cell Signaling), RB p-Ser612 mAb (4E4, Abnova), Chk2 (H-300, Santa Cruz), Chk2 p-Thr68 (#2661, Cell Signaling) and E2F1 Ab (C-20, Santa Cruz). To perform co-immunoprecipitation assays, 600 µg of Saos-2 cell extract was prepared in NP-40 lysis buffer and pre-cleared with 100 µl of 20% protein A-Sepharose beads (Pharmacia) for 1.5 h at 4°C. To immunoprecipitate RB-associated E2F1, 500 µg of lysates prepared above were incubated with anti-RB antibody at 4°C overnight. Then 150 µl of 20% protein A-Sepharose beads were added to pull down the immunocomplexes for 3 h at 4°C. The beads were washed with NP-40 lysis buffer four times and resuspended in 4X SDS sample buffer. The immunocomplexes were resolved in 10% SDS-PAGE and analyzed with immunoblotting using anti-RB and anti-E2F1 antibodies.

### Yeast Strains and Yeast Plasmids

*S. cerevisiae cdc28-4* [21] and *cdc28-13* [21] were gifts from Dr. Curt Wittenberg (Department of Molecular Biology, The Scripps Research Institute, CA). Both strains were cultured in YPD (1.0% yeast extract, 2.0% bacto peptone, 20% glucose) medium. pVTU101 (from Dr. Shu-Chun Teng, Graduate Institute and Department of Microbiology, National Taiwan University) based plasmids expressing BGLF4, K102I, HA-Cdc2 and UL97 (pMTS1, pMTS2, pMTS3 and pMTS4) were generated by cloning a BamHI-BGLF4 fragment from pYPW17, BamHI-K102I fragment from pYPW20, XbaI-HA-Cdc2 fragment partially digested from pSMcdc2H-HATag (a gift from Dr. David Morgan (Department of Physiology and Biochemistry, University of California, San Francisco, CA), and a HindIII/XbaI fragment of HCMV UL97 from pTAG-UL97. pMTS5 and pMTS7 are constructs expressing HA-tagged BGLF4 and UL97, generated by inserting an HA linker into the BamHI site of pMTS1 and HindIII site of pMTS4.

### Spot Dilution Assay

*S. cerevisiae cdc28-4* cells containing various plasmids as indicated were adjusted to an OD600 to 0.5, and diluted serially 5-fold in sterile water. Two µl of each dilution were spotted onto YPD plates. The plates were incubated at 23, 30 or 37°C for 2 days.

### Yeast Budding Assay and FACS Analysis

*S. cerevisiae cdc28-13* cells were grown at 23°C in SC medium (0.17% yeast nitrogen base without amino acids, 0.5% ammonium sulfate, 0.2% amino acid drop-out mix, 20% dextrose) and lacking uracil to 0.5 OD at 600 nm. Hydroxyurea (HU, 200 mM) was added into the culture medium and incubated at 23°C for 3 h. Then, cells were collected and quickly resuspended in fresh YPD medium without HU at 38°C for 4 h. For budding assays, cells were fixed in 3.7% (vol/vol) formaldehyde at 4°C overnight. Cell aggregates were sonicated and counted under phase contrast microscopy (Axioskop 49 FL, Zeiss) for the percentage of budded cells. Before FACS analysis, 10^7^ yeast cells were fixed in 70% ethanol at −20°C overnight, washed with PBS and treated with 50 mM Tris-HCl containing RNase A (100 µg/ml) at 37°C for 1 h. Cells were stained with 10 µg/ml PI (propidium iodide) at 4°C overnight and the DNA contents were then analyzed by flow cytometry.

### Indirect Immunofluorescence Assay

At the time points indicated post transfection or induction, slide-cultured cells were fixed with 4% paraformaldehyde at room temperature for 20 min and washed with PBS for 5 min. Cells were permeabilized with 0.1% Triton X-100 for 5 min and subjected to immunofluorescence staining. Cells were first stained with anti-BGLF4 mAb (2224) at 37°C for 1.5 h, followed by fluorescein isothiocyanate-conjugated anti-mouse immunoglobulin G (IgG) (Jackson) or rhodamine red-conjugated anti-mouse IgG (Jackson) antibodies at 37°C for 1 h. DNA was stained with Hoechst 33258 at room temperature (5 min for 293 T-REx inducible cells or 90 sec for HeLa and NPC-TW01 cells). Slides were mounted with mounting medium (H1000, Vector) and observed by fluorescence microscopy (Axioskop 49 FL, Ziess).

### Time-lapse Microscopy

To capture fluorescence images of living cells, GFP-H2B-HeLa cells were seeded into 6-well plates and transfected with DsRed-BGLF4, DsRed-K102I or DsRed expressing plasmids. At 6 h post transfection, fields of view were selected and cells were subjected to monitoring every 10 min over a 18 h duration under an automated inverted fluorescence microscope (Axiovert 200M, Zeiss). Images were captured by Metamorph software (Universal Imaging). During imaging, cells were supplemented with 5% CO_2_ and a heat stage was used to maintain them at 37°C.

### MTT Assay

To measure cell proliferation, 1×10^3^ 293 T-REx inducible cells or 2×10^3^ NPC-TW01 T-REx inducible cells in 200 µl medium were seeded into 96-well plates in triplicate with or without Dox (100 ng/ml for 293 T-REx or 50 ng/ml for NPC-TW01 T-REx). An MTT assay was performed every 24 h after cell seeding. In brief, the medium was aspirated and replenished with fresh medium containing MTT (1 mg/ml, Sigma). After incubation at 37°C with 5% CO_2_ for 4 h, the medium with MTT was removed and 100 µl DMSO was added to dissolve formazan crystals. The 96-well plates were shaken for 10 min at room temperature to make sure all the crystals were completely dissolved. Then absorbance was detected at 550 nm using an ELISA reader. The percentage of cell proliferation against the control was calculated by dividing the absorbance of cells treated with Dox by the absorbance of cells without Dox treatment.

### Analysis of Micronucleus Formation

To analyze the formation of micronuclei (MN), slide-cultured 293 T-REx V4, B22 and K9 cells or NPC-TW01 T-REx VIT7 and KIT2 cells were incubated with Dox for 72 h. Cells were fixed with 4% paraformaldehyde at room temperature for 20 min and stained with Hoechst33258 for 5 min. Micronuclei were examined using fluorescence microscopy (Axioskop 49 FL, Ziess). At least 1000 cells were counted for each experiment.

### Reporter Assay

To investigate whether BGLF4 regulates cyclin D1and ZBRK1 promoter activity, 5×10^5^ Saos-2 cells were seeded into 6-well plates and transfected with reporter gene plasmid CCND1-P or ZBRK1-B along with expression plasmids as indicated. The amount of total DNA was kept constant by supplementing vector pEGFP-C1. pWP1 (pCMV-*Renilla reniformis)* was co-transfected in each reaction for normalizing transfection efficiencies. Luciferase activity was determined with the dual-luciferase assay system (Promega) at 48 h post transfection.

## Supporting Information

Figure S1 **Comparison of BGLF4 expression levels in various cell lines.** NA cell line is a recombinant Akata EBV-converted NPC-TW01 line. EREV8 and Maxi EBV cell line are derived from 293TREx_Flag_ERTA cell line. EREV8 cells were generated by infecting 293TREx_Flag_ERTA with Akata EBV by cocultivation with EBV-producing B cells. 293-Maxi EBV harbors the EBV bacmid Maxi. NA cells were reactivated with 40 ng 12-O-tetradecanoylphorbol-13-acetate-3 mM sodium butyrate (TPA/SB) for 24 h. EREV8 and Maxi EBV was induced with 100 ng/ml Dox for 24 h for the expression of Rta to induce lytic cycle progression. B22 is a 293 T-REx BGLF4 inducible cell line, which was induced with100 ng/ml Dox for 24 h for BGLF4 expression. HeLa cells were seeded at a concentration of 2×10^6^ cells per 10-cm dish and transfected with 5 µg GFP-BGLF4 and harvested at 24 h post transfection. The protein expression levels of BGLF4 were resolved by 10% SDS-PAGE and immunoblotted with specific antibodies. GAPDH served as a loading control.
(TIF)

Figure S2 **The kinase activity of viral protein kinase in** ***S.cerevisiae cdc28-13*** **.** (A) *S.cerevisiae cdc28-13* with protein expression as indicated was cultured in 10 ml Ura-SC broth to an OD at 600 nm of 1.0. The yeast extracts were collected for IP-kinase assay. EBV BGLF4, HCMV UL97 and human Cdc2 were immunoprecipitated with HA antibody (HA.11, Covance). The precipitated proteins were detected using HA antibody. (B) For *in vitro* kinase assay, the immunoprecipitates were incubated with kinase buffer (20 mM Tris-HCl, 1 mM EDTA, 1 mM DTT, 10 mM MgCl_2_, 0.2 mM Na_3_VO_4_, 100 mM ATP) containing[/−^32^P]ATP with 1 µg histone H1 (Calbiochem) at 30°C for 30 min. After kinase reaction, proteins were resolved by 12% SDS-PAGE. Gels were dried and subjected to autoradiography for 12 h.
(TIF)

Figure S3 **Expression kinetics of BGLF4 in 293 T-REx BGLF4 inducible cells.** (A) Various 293 T-REx BGLF4 inducible clones, B9, B10, B17, B19, B20 and B22, were treated with 100 ng/ml doxycycline (Dox) for 24 h. The BGLF4 and GAPDH proteins were resolved by 10% SDS-PAGE and immunoblotted with specific antibodies. GAPDH served as a loading control. (B) B22 cells were induced with 100 ng/ml Dox for the times indicated. The proteins were displayed by SDS-PAGE and detected with specific antibodies. (C) Slide cultured B9, B20 and B22 cells were incubated with 100 ng/ml Dox. At 24 h post induction (hpi), cells were fixed with 4% paraformaldehyde and stained for BGLF4 with monoclonal antibody 2224 and DNA with Hoechst 33258. (D) Slide cultured B22 cells were incubated with 100 ng/ml Dox, harvested at the time points indicated and stained for BGLF4 and DNA. Chromosome condensation was observed at 6 hpi in B22 cells. (E) B9, B20 and B22 cells were seeded in 96-well plate in a triplicate manner and induced with 100 ng/ml Dox for the expression of BGLF4. At 24, 48, 72, 96 and 120 hpi, an MTT assay was performed and the optical densities (OD) were determined by spectrophotometry at 550 nm.
(TIF)

Figure S4 **Expression kinetics of BGLF4 in NPC-TW01 T-REx BGLF4 inducible cells.** (A) The NPC-TW01 T-REx BGLF4 inducible clones KIT1, KIT2, KIT3, KIT20, KIT21 and KIT22 were treated with 50 ng/ml doxycycline (Dox) for 24 h. The BGLF4 protein was resolved by 10% SDS-PAGE and immunoblotted with specific antibodies. GAPDH served as a loading control. (B) KIT2 cells were induced with 50 ng/ml Dox and cell extracts were collected at the time points indicated. The protein expression was displayed by SDS-PAGE and detected with specific antibodies. (C) Slide cultured KIT2 and KIT21 cells were incubated with 50 ng/ml Dox. At 60 h post induction (hpi), cells were fixed with 4% paraformaldehyde and stained for BGLF4 with monoclonal antibody 2224 and DNA with Hoechst 33258. More than 95% of the cells expressed BGLF4.
(TIF)
