# Supplementary figures and images for: Epstein-Barr Virus BGLF4 Kinase Retards Cellular S-Phase Progression and Induces Chromosomal Abnormality
Source: PLoS One. 2012 Jun 29;7(6):e39217. doi: 10.1371/journal.pone.0039217 (PMC3387188; doi:10.1371/journal.pone.0039217)

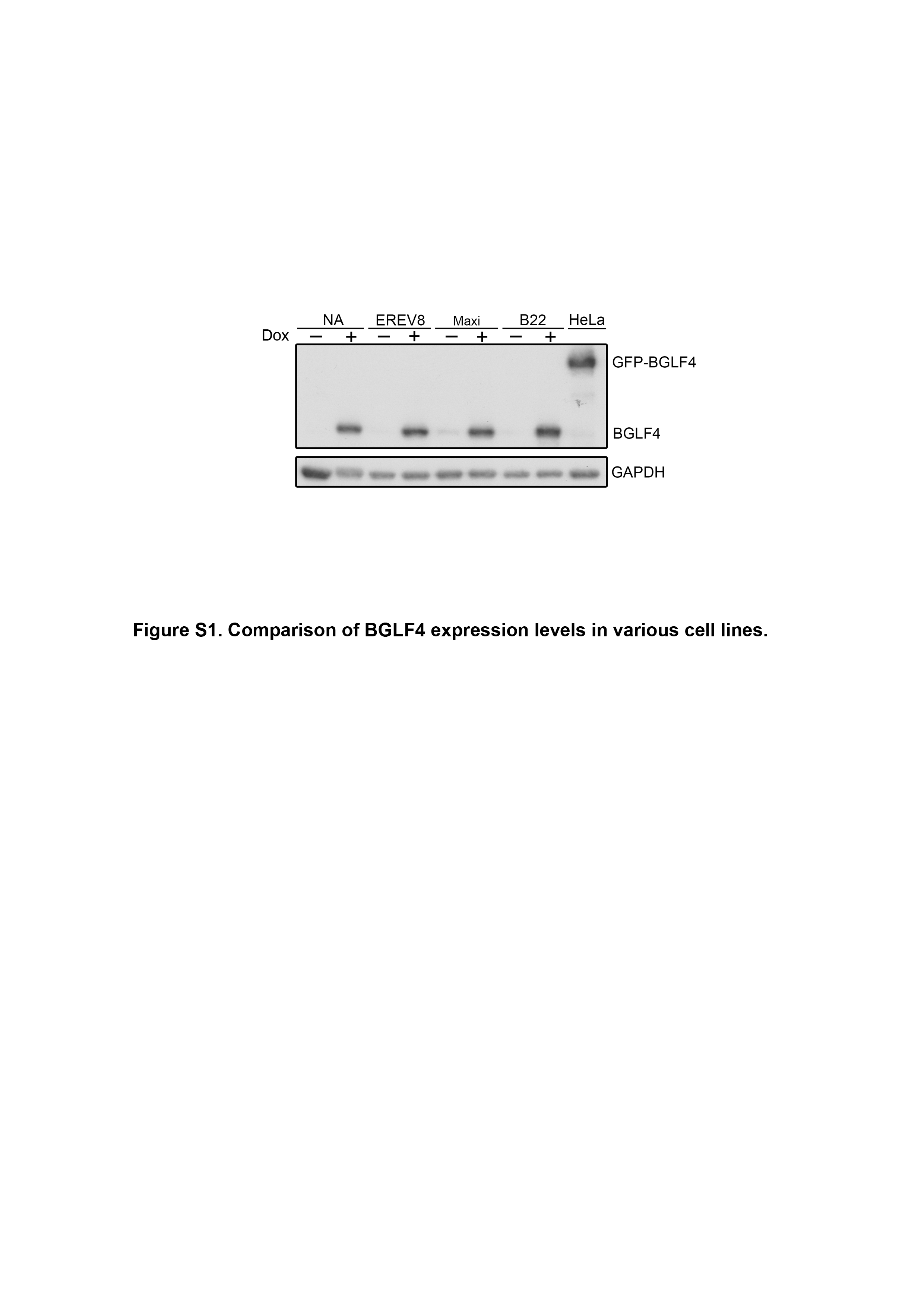

Supplement: Figure S1 — Comparison of BGLF4 expression levels in various cell lines. NA cell line is a recombinant Akata EBV-converted NPC-TW01 line. EREV8 and Maxi EBV cell line are derived from 293TREx_Flag_ERTA cell line. EREV8 cells were generated by infecting 293TREx_Flag_ERTA with Akata EBV by cocultivation with EBV-producing B cells. 293-Maxi EBV harbors the EBV bacmid Maxi. NA cells were reactivated with 40 ng 12-O-tetradecanoylphorbol-13-acetate-3 mM sodium butyrate (TPA/SB) for 24 h. EREV8 and Maxi EBV was induced with 100 ng/ml Dox for 24 h for the expression of Rta to induce lytic cycle progression. B22 is a 293 T-REx BGLF4 inducible cell line, which was induced with100 ng/ml Dox for 24 h for BGLF4 expression. HeLa cells were seeded at a concentration of 2×10^6^ cells per 10-cm dish and transfected with 5 µg GFP-BGLF4 and harvested at 24 h post transfection. The protein expression levels of BGLF4 were resolved by 10% SDS-PAGE and immunoblotted with specific antibodies. GAPDH served as a loading control. (TIF) [file pone.0039217.s001.tif]

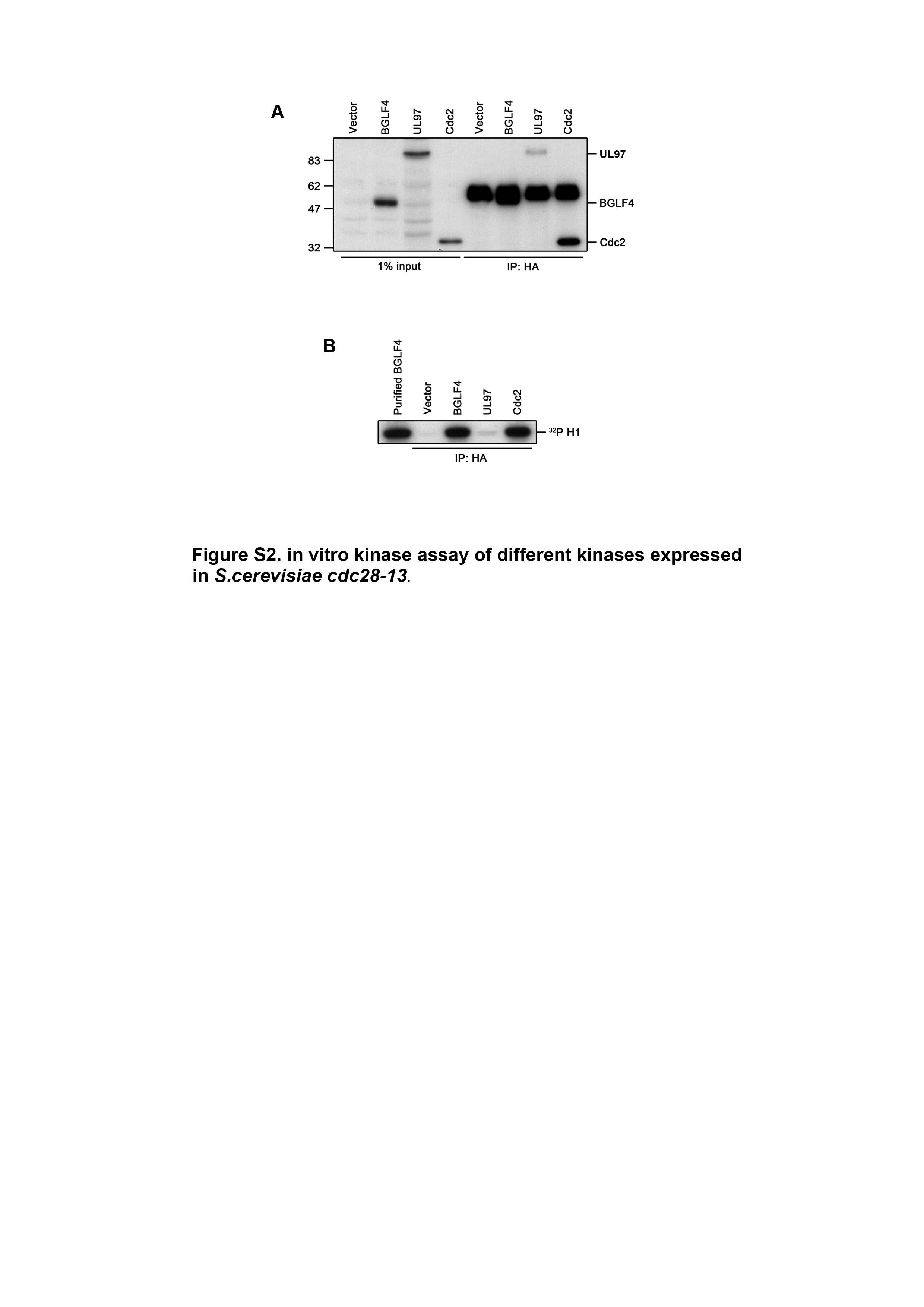

Supplement: Figure S2 — The kinase activity of viral protein kinase in S.cerevisiae cdc28-13 . (A) S.cerevisiae cdc28-13 with protein expression as indicated was cultured in 10 ml Ura-SC broth to an OD at 600 nm of 1.0. The yeast extracts were collected for IP-kinase assay. EBV BGLF4, HCMV UL97 and human Cdc2 were immunoprecipitated with HA antibody (HA.11, Covance). The precipitated proteins were detected using HA antibody. (B) For in vitro kinase assay, the immunoprecipitates were incubated with kinase buffer (20 mM Tris-HCl, 1 mM EDTA, 1 mM DTT, 10 mM MgCl2, 0.2 mM Na3VO4, 100 mM ATP) containing[/−32P]ATP with 1 µg histone H1 (Calbiochem) at 30°C for 30 min. After kinase reaction, proteins were resolved by 12% SDS-PAGE. Gels were dried and subjected to autoradiography for 12 h. (TIF) [file pone.0039217.s002.tif]

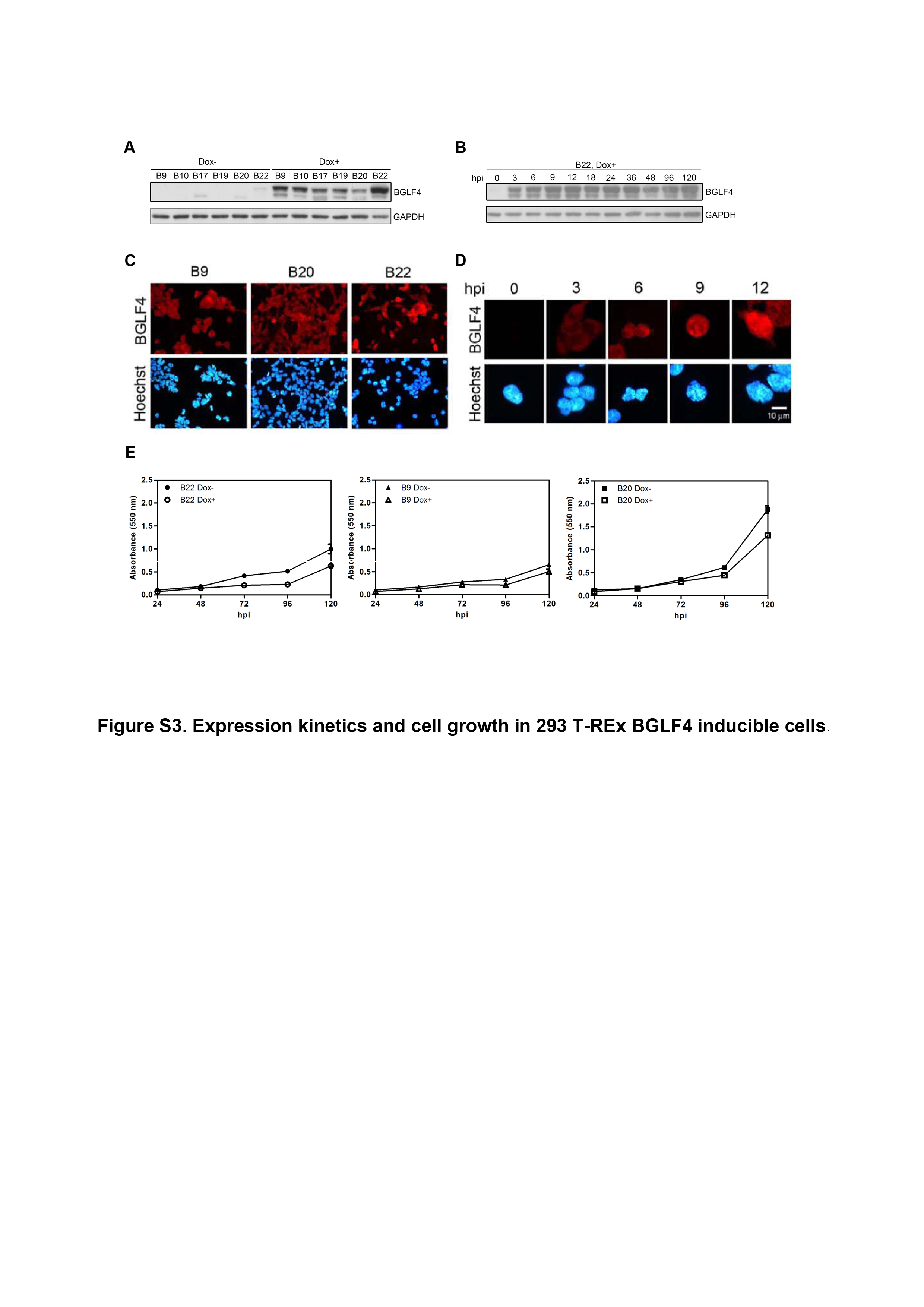

Supplement: Figure S3 — Expression kinetics of BGLF4 in 293 T-REx BGLF4 inducible cells. (A) Various 293 T-REx BGLF4 inducible clones, B9, B10, B17, B19, B20 and B22, were treated with 100 ng/ml doxycycline (Dox) for 24 h. The BGLF4 and GAPDH proteins were resolved by 10% SDS-PAGE and immunoblotted with specific antibodies. GAPDH served as a loading control. (B) B22 cells were induced with 100 ng/ml Dox for the times indicated. The proteins were displayed by SDS-PAGE and detected with specific antibodies. (C) Slide cultured B9, B20 and B22 cells were incubated with 100 ng/ml Dox. At 24 h post induction (hpi), cells were fixed with 4% paraformaldehyde and stained for BGLF4 with monoclonal antibody 2224 and DNA with Hoechst 33258. (D) Slide cultured B22 cells were incubated with 100 ng/ml Dox, harvested at the time points indicated and stained for BGLF4 and DNA. Chromosome condensation was observed at 6 hpi in B22 cells. (E) B9, B20 and B22 cells were seeded in 96-well plate in a triplicate manner and induced with 100 ng/ml Dox for the expression of BGLF4. At 24, 48, 72, 96 and 120 hpi, an MTT assay was performed and the optical densities (OD) were determined by spectrophotometry at 550 nm. (TIF) [file pone.0039217.s003.tif]

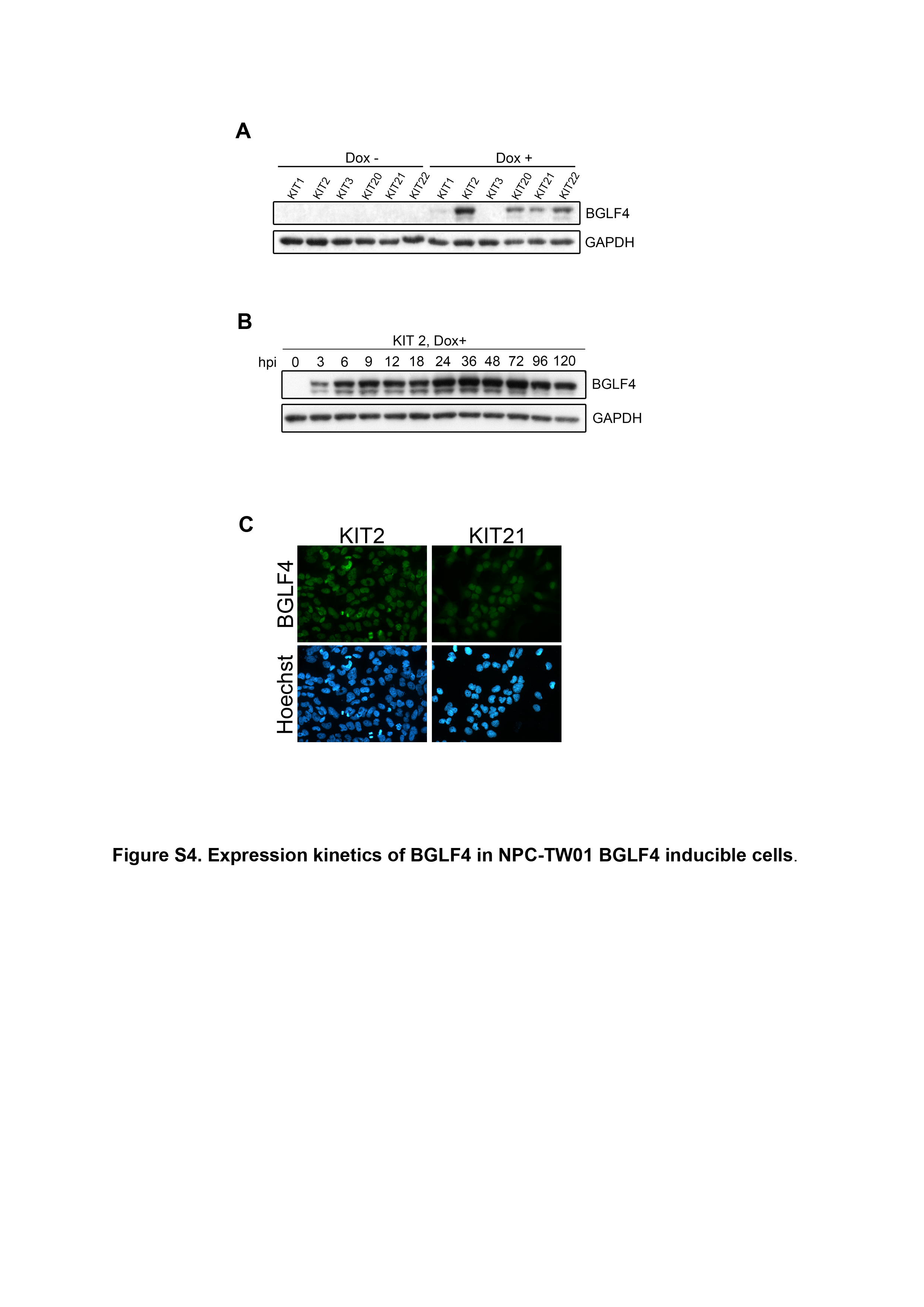

Supplement: Figure S4 — Expression kinetics of BGLF4 in NPC-TW01 T-REx BGLF4 inducible cells. (A) The NPC-TW01 T-REx BGLF4 inducible clones KIT1, KIT2, KIT3, KIT20, KIT21 and KIT22 were treated with 50 ng/ml doxycycline (Dox) for 24 h. The BGLF4 protein was resolved by 10% SDS-PAGE and immunoblotted with specific antibodies. GAPDH served as a loading control. (B) KIT2 cells were induced with 50 ng/ml Dox and cell extracts were collected at the time points indicated. The protein expression was displayed by SDS-PAGE and detected with specific antibodies. (C) Slide cultured KIT2 and KIT21 cells were incubated with 50 ng/ml Dox. At 60 h post induction (hpi), cells were fixed with 4% paraformaldehyde and stained for BGLF4 with monoclonal antibody 2224 and DNA with Hoechst 33258. More than 95% of the cells expressed BGLF4. (TIF) [file pone.0039217.s004.tif]
